# Accentuated eccentric loading in lower-body resistance training: a systematic review of acute and chronic adaptations on strength, power, and speed outcomes

**DOI:** 10.3389/fphys.2025.1720205

**Published:** 2026-01-30

**Authors:** Jinghui Zhong, Tongwu Yu, Yan Xiao, Hao Wu

**Affiliations:** 1 Capital University of Physical Education and Sports, Beijing, China; 2 Anhui Communications Vocational and Technical College, Hefei, Anhui, China

**Keywords:** accentuated eccentric loading, eccentric-to-concentric ratio, post-activation potentiation enhancement, resistance training, strength–power

## Abstract

**Background and Objectives:**

Accentuated eccentric loading (AEL) prescribes an eccentric load exceeding the paired concentric load, exploiting the muscle’s greater force capacity during lengthening. Evidence suggests benefits, but findings on its acute and chronic efficacy *versus* traditional resistance training (TRT) remain inconsistent. Uncertainties persist regarding acute potentiation, optimal eccentric-to-concentric ratios, and transfer to sport performance. This review synthesises current evidence, distinguishing acute from chronic outcomes and summarising prescription variables to guide practice.

**Methods:**

This review followed PRISMA 2020 guidelines. PubMed, Scopus, Web of Science, and Embase were searched to June 2025 for acute and chronic trials where eccentric loading exceeded concentric loading in lower limb exercises. We included peer-reviewed acute and longitudinal trials in healthy humans that (i) applied Accentuated eccentric loading to lower-body resistance or jump exercises, (ii) compared AEL with a traditional isoinertial or equal-load condition, and (iii) reported at least one outcome related to maximal strength, jump performance, sprint speed or change-of-direction ability. Data on study design, AEL configuration (eccentric and concentric loads, movement type, loading method, volume and frequency) and performance outcomes were extracted. Standardized effect sizes were taken from the original articles where reported and summarized qualitatively; no new meta-analytic pooling was performed due to heterogeneity in study designs and incomplete reporting.

**Results:**

Twenty trials met the inclusion criteria. Acute studies showed that AEL enhanced explosive performance when eccentric intensity was set at 110%–120% of concentric one-repetition maximum (1RM) in back squats or when an additional 10%–30% of body mass was applied in dumbbell release jump drills. However, responses were variable, with very high loads or poor timing occasionally impairing performance. Chronic training programs reported maximal strength gains ranging from approximately 9%–22%, with jump height improvements between 4% and 11%. While these adaptations were generally superior or comparable to traditional training, the magnitude of transfer to sprint and COD performance was inconsistent. Certainty of evidence (GRADE) was moderate for strength outcomes due to consistent positive effects, but low to very low for speed outcomes due to imprecision and heterogeneity.

**Conclusion:**

AEL is an effective method to enhance lower body maximal strength and explosive performance, particularly when applying 110%–120% 1RM in multi-joint lifts or adding 10%–30% body mass in jump drills. However, the transfer of these adaptations to sprint and change-of-direction speed remains uncertain. Due to the high heterogeneity of study protocols and small sample sizes, these findings should be interpreted with caution. Future research requires standardized reporting and larger randomized trials to optimize programming.

## 1 Introduction

The ability to generate high levels of lower limb power is fundamental to decisive actions in sport, including sprint acceleration, vertical jumping, and rapid changes of direction (Suchomel et al., 2018). Meta-analytic evidence indicates that gains in maximal lower body strength can translate into faster sprint times, yet the transfer is inconsistent, suggesting that conventional approaches may not fully optimize explosive outcomes (Seitz et al., 2014). Traditionally, research has sought to identify an “optimal power load” (Cormie et al., 2007; Buskard et al., 2018; Hoffman et al., 2005), but contemporary reviews emphasize that this relationship is exercise-specific and strongly moderated by training status (Suchomel et al., 2018; Cormie et al., 2011; Kawamori and Haff, 2004).

A key limitation of traditional resistance training (TRT) is the use of symmetrical loading (Androulakis-Korakakis et al., 2023; Toji and Kaneko, 2004). Since skeletal muscle exhibits 40%–50% greater force capacity during eccentric than concentric contractions (Nuzzo et al., 2023a; Hollander et al., 2007), TRT loads prescribed relative to concentric strength may underutilise the eccentric contribution to the stretch-shortening cycle (SSC) (Hollander et al., 2007; Douglas et al., 2017a). Accentuated eccentric loading (AEL) addresses this by prescribing an eccentric load that exceeds the concentric load (>100% 1RM or added mass during the eccentric phase) (Wilson et al., 1993; Merrigan et al., 2022). This differs conceptually from simply slowing repetition tempo to increase time under tension (Douglas et al., 2018); AEL specifically targets the magnitude of the mechanical stimulus.

The application of AEL aims to elicit acute or chronic enhancements. Acutely, AEL can transiently enhance concentric velocity through post-activation performance enhancement (PAPE), although this effect is sensitive to fatigue management (Douglas et al., 2018). Chronically, interventions lasting several weeks suggest gains in maximal strength and power that are superior or comparable to TRT (Walker et al., 2016; Douglas et al., 2017b). However, findings remain inconsistent, with some studies reporting limited transfer to sport-specific actions like sprinting (Suarez-Arrones et al., 2018).

Uncertainties persist regarding the optimal programming parameters–specifically the eccentric intensity, the eccentric-to-concentric ratio, and the influence of training status (Seitz and Haff, 2016; Rappelt et al., 2024). Furthermore, previous reviews have not systematically distinguished acute responses from chronic adaptations while collating these prescription variables to inform practice (Merrigan et al., 2022).

Therefore, the aim of this systematic review is to synthesise the acute and chronic effects of lower-body AEL on maximal strength, power, sprint, and change-of-direction (COD) outcomes. Using the PICOS framework, we specifically examine: (i) Population: Healthy adults; (ii) Intervention: AEL where eccentric load objectively exceeds concentric load; (iii) Comparator: Traditional isoinertial or equal-load training; (iv) Outcomes: Maximal strength, vertical jump height, sprint speed, and COD ability; and (v) Study Design: Acute and longitudinal trials.

## 2 Methods

### 2.1 Registration of systematic review protocol

This systematic review was conducted in accordance with the methodological framework outlined in the Cochrane Handbook for Systematic Reviews of Interventions (Version 6.0) and reported following the 2020 Preferred Reporting Items for Systematic Reviews and Meta-Analyses (PRISMA) guidelines (Page et al., 2021). The protocol was prospectively registered in the INPLASY database under registration number INPLASY202560101. Given the anticipated heterogeneity in study designs and outcome measures, a narrative synthesis approach was predefined in the protocol. Meta-analytical procedures were considered only for subgroups of studies with high methodological and outcome homogeneity. As specified in the protocol, a meta-analysis was not conducted since the available studies were highly heterogeneous in design (few RCTs and many crossover or quasi experimental trials), exercise protocols (eccentric load magnitude, concentric comparison loads, release timing), and outcome definitions (strength, power, and speed assessed with different units). In addition, the number of high quality RCTs was insufficient to support a robust pooled estimate. We therefore adopted a structured narrative synthesis.

### 2.2 Eligibility criteria

Using the PICOS framework (Schardt et al., 2007), studies were assessed for inclusion and exclusion according to the following criteria. Exclusion criteria: (i) Participants presenting with any musculoskeletal pathology or limb impairments, whether congenital or training induced. (ii) Studies using training protocols that did not meet the operational definition of AEL. Specifically, this included: traditional isoinertial training where the eccentric load was equal to the concentric load; studies where the magnitude of eccentric overload was not quantified; and flywheel-based training, as the coupled nature of inertial resistance prevents the independent manipulation of eccentric intensity relative to the concentric phase (Wagle et al., 2017). (iii)Non-English language publications. (iv) Publications classified as literature reviews, meta-analyses, dissertations, abstracts, conference proceedings, or case reports. Eligibility criteria for study inclusion were established based on the PICOS framework, as follows:(i) Population: Healthy adults, including trained athletes, recreationally active individuals, or untrained participants. All participants had to be free of musculoskeletal disorders or injuries at the time of testing. (ii) Intervention: AEL, defined as a resistance training protocol in which the eccentric phase was loaded more heavily than the concentric phase. Acceptable implementations included external weights, manual overload assistance, or other methods of eccentric force enhancement. The comparison condition was either conventional resistance training with symmetrical loading or a baseline/control condition in acute trials. (iii) Outcomes: At least one of the following performance outcomes: maximal lower limb strength (e.g., one repetition maximum), explosive performance (e.g., jump height, peak power, movement velocity), or speed related performance (e.g., sprint time or change of direction). (iv) Study design: Randomized controlled trials (parallel group or crossover) and other experimental trials with valid control conditions, with pre- and post-intervention quantitative data or within-subject comparisons in acute experiments. (v) Study type and language: Only full text, peer-reviewed original research articles published in English. This review focused exclusively on interventions where eccentric loading was explicitly set higher than concentric loading (Wagle et al., 2017). Eligible studies had to deliberately accentuate eccentric demand in multi-joint resistance exercises or jump drills, with the eccentric stimulus clearly quantified. Minimum standards included a defined eccentric intensity relative to a concentric reference (e.g., % of 1RM or % of body mass in eccentric drills), a specified release timing of the additional load, and sufficient total exposure to elicit a training response. Studies lacking adequate detail on these parameters were excluded. To be classified as AEL, an intervention had to meet the following criteria: (i) the external load applied during the eccentric phase of each repetition exceeded the load used during the concentric phase of the same repetition (weight releasers, elastic bands or computer-controlled resistance); or (ii) in jump or plyometric drills, an additional external load was applied during the downward countermovement and removed before take-off so that the concentric phase was performed with a lighter load. Studies that only manipulated eccentric tempo, range of motion, or time under tension while keeping eccentric load equal to concentric load were not considered AEL and were therefore excluded.

### 2.3 Information sources and search strategy

A comprehensive literature search was conducted across four major electronic databases: PubMed, Scopus, Web of Science, and Embase, covering all records up to June 2025. The search strategy combined controlled vocabulary such as MeSH terms with free text keywords. Terms related to eccentric overload training included “eccentric overload”, “accentuated eccentric”, “enhanced eccentric”, and “eccentric-concentric”. These were systematically combined with performance related terms such as “strength”, “power”, “explosive”, “velocity”, “jump,” and “performance”. Peer reviewed full text original articles published in English were eligible. To enhance the comprehensiveness and sensitivity of the search, the Word Frequency Analyser was utilized to identify additional relevant keywords. The Research Refiner tool was employed to optimize the balance between sensitivity and specificity in the PubMed search. Furthermore, the Polyglot Search Translator was used to adapt the finalized PubMed search strategy for other databases.

To identify unpublished or ongoing studies, we searched international trial registries, including the WHO International Clinical Trials Registry Platform (ICTRP) and ClinicalTrials.gov. Reference lists of all included studies and relevant systematic reviews were manually screened, and forward citation tracking was conducted in Google Scholar. Finally, automated alerts were set up to capture new publications until the final update in June 2025.

### 2.4 Study selection

Two reviewers (ZJH and YTW) independently screened titles, abstracts, and full texts, with disagreements resolved by consensus. If consensus was not achieved, a third reviewer (XY) adjudicated. Initially, all duplicate records were removed using EndNote reference management software (version X9.0.3; Clarivate Analytics, Philadelphia, PA, United States). Titles and abstracts were then screened to assess eligibility, and full texts of potentially relevant articles were retrieved and evaluated against the predefined inclusion and exclusion criteria. The screening process was conducted using Rayyan, a web based systematic review platform. Studies were excluded if they were reviews, case reports, uncontrolled observational designs, or conference abstracts lacking sufficient methodological detail.

### 2.5 Data extraction

Data extraction was conducted using a structured spreadsheet and independently cross verified by two reviewers (Z.J.H. and Y.T.W.). A calibrated form was used to capture participant characteristics, exercise mode, eccentric and concentric loading parameters, release timing, frequency, volume, outcome definitions, and testing protocols. For each study, ES and significance levels comparing AEL groups with control or conventional training groups were recorded. When data were not directly reported in the manuscripts, WebPlotDigitizer (https://automeris.io/WebPlotDigitizer/) was used to extract numerical values from figures. Corresponding authors were contacted to request missing data (e.g., means and standard deviations not reported in text or figures); however, no additional raw data were provided. Consequently, where descriptive statistics could not be reliably extracted or calculated, findings were described qualitatively rather than attempting data imputation. Any reported adverse events or practical implementation challenges were also documented. Where available, standardized ES reported in the original articles were extracted and summarized in the acute and chronic overview Tables 1, 2. When ES were not reported and the necessary descriptive statistics were unavailable, new ES were not calculated and the findings were described qualitatively.

**TABLE 1:** Acute AEL training studies.

Study	Movement	Loading method	Protocol		Assessment and timing	Results	Conclusions
			E	C			
			Load magnitude	Load magnitude			
Aboodarda et al. (2014)	Drop jump	Elastic bands	CON: BW ECC: 20/30% BW	CON: BW ECC: BW	DJ Takeoff velocity Immediate (Same rep)	DJ height 20 cm: no change in DJ20 (0.0%) and a slight decrease in DJ30 (−2.4%) 35 cm: small improvements observed in both DJ20 (+2.5%) and DJ30 (+2.5%) 50 cm: moderate increases for DJ20 (+2.9%) and DJ30 (+2.6%). Takeoff velocity 20 cm: slight reductions in DJ20 (−0.4%) and DJ30 (−0.7%) 35 cm: minimal change in DJ30 (0%) and a slight increase in DJ20 (+0.4%). 50 cm: notable improvement in both DJ20 and DJ30 (+1.1%) ES: trivial-moderate effects	Minor to moderate performance increases at higher drop heights (35–50 cm). Non-significant or negative effects at 20 cm. No consistent group superiority across conditions. DJ30 showed slightly more sensitivity to lower drop heights, while both conditions benefited similarly from 50 cm
Aboodarda et al. (2013)	CMJ	Elastic bands	CON: BW ECC: 20/30% BW	CON: BW ECC: BW	CMJ PV PF PP Immediate (Same rep)	CMJ20 showed a moderate increase (+5.3%), while CMJ30 yielded a large improvement (+10.5%) PV: No change in CMJ20 (0%), but a noticeable increase in CMJ30 (+16.7%) PF: Minimal increase in CMJ20 (+0.6%), modest improvement in CMJ30 (+2.9%) PP: Moderate gain in CMJ20 (+6.4%), substantial improvement in CMJ30 (+30.2%) ES: moderate-to-large	Greater enhancements observed in CMJ30 across all metrics, especially in PV and PP. AEL30 condition led to superior performance gains compared to AEL20, suggesting load intensity-dependent effects.
Bridgeman et al. (2017b)	Drop jump	Dumbbells	CON: BW ECC: 10/20/30% BW	CON: BW ECC: BW	DJ CMJ PP 2, 6, and 12 min	DJ: BW condition showed greater improvement than 10% and 30% BW (ES: 0.39, 0.34) 20% BW load outperformed both 10% and 30% BW (ES:0.37, 0.32) CMJ: 20% BW condition yielded greater gains than BW, 10%, and 30% BW loads PP: 20% BW significantly greater than BW, 10%, and 30% BW conditions ES = 0.32–0.39 (small to moderate)	20% BW consistently produced superior performance outcomes in CMJ and PP. BW condition slightly outperformed higher loads in drop jump. Non-linear response pattern suggests an optimal eccentric overload exists around 20% BW for maximizing explosive performance.
Moore et al. (2007)	Jump squat	Weight releaser	CON: 30% 1RM ECC: 50/80/110% 1RM	CON: 30% 1RM ECC: 30% 1RM	PV PF PP Immediate (Same rep)	PV:Slight decreases at ECC50% and ECC80% (both −0.14); minimal increase at ECC110% (+0.05) PF:Negligible change at ECC50% (+0.01); slight reductions at ECC80% (−0.08) and ECC110% (−0.09) PP:Minimal change at ECC50% (+0.02) and ECC80% (0.00); moderate increase at ECC110% (+0.14) PV, PF, PP: ES = −0.14 to 0.14 (trivial to small)	ECC110% elicited the most favorable response in PP and PV despite reductions in PF. ECC50% and ECC80% showed limited or negative effects across metrics. Suggests that supramaximal eccentric loading (110%) may be more effective for enhancing power output
Sheppard et al. (2007)	CMJ	Dumbbells	CON: BM ECC: 20% BM	CON: BW ECC: BW	Jump height PV PF PP Immediate (Same rep)	Jump height: Slight improvement under AEL (+4.3%, ES = 0.20 (small)); BJ:48.4 cm, AEL: 50.5 cm PV: Moderate increase with AEL (+9.4%, ES = 0.39); BJ: 4,655.9, AEL: 5,095.5 PF:Small increase (+3.9%, ES = 0.19); BJ: 2,181.4, AEL: 2,265.9 PP: Small improvement (+3.1%, ES = 0.25); BJ: 2.8, AEL: 2.9.	AEL elicited modest to moderate enhancements in jump performance metrics, particularly in PV. All variables favored AEL over BJ, though effect sizes remained small to moderate
Wagle et al. (2018)	squat	Weight releaser	CON: 80% 1RM ECC: 105% 1RM	CON: 80% 1RM ECC: 80% 1RM	PP CON RFD ECC RFD MV Intra-set (Reps 1, 3, 5)	PP Slightly higher in AEL (2,704.62) than TRT (2,638.12) CON RFD TRT: Moderate improvement with AEL (1704.26 vs. 1,518.94) ECC RFD: AEL showed a noticeable increase (2,766.49) compared to TRT (2,515.93) Mean velocity: No difference observed (both 0.54 m/s)	AEL resulted in moderate improvements in both CON and ECC RFD, as well as a small increase in PP, while mean movement velocity remained unchanged. Suggests neuromuscular benefits of AEL over TRT, especially in force development
Merrigan et al. (2020)	squat	Weight releaser	CON:65/80% 1RM ECC:120% 1RM 65% 1RM: 3 × 5 80% 1RM: 3 × 3 AEL on first rep	CON: 65/80% 1RM ECC: 65/80% 1RM 65% 1RM: 3 × 5 80% 1RM: 3 × 3	CON/ECC MV CON/ECC PV CON/ECC MPCON/ECC pp CON MF CON PF Intra-set (Reps 1–5)	ECC MV: AEL65>TRT65 ECC PV: AEL65>TRT65 AEL65 > 80 CON PF: AEL65>AEL80>TRT80 ECC MP: AEL65>TRT65 AEL80>TRT80 ECC PP: AEL65>TRT65 AEL80>TRT80	AEL65 consistently outperformed TRT65 and AEL80 across eccentric velocity and power metrics. Concentric force output showed a descending trend from AEL65 to TRT80, indicating superior neuromuscular output at moderate eccentric load (65%).
Munger et al. (2017)	squat	Weight releaser	CON: 90% 1RM ECC: 105%/110%/120% 1RM	-	pGRF PP RFD PV Immediate (Same rep)	pGRF: 120% > 110%>105% PP: 120% > 110%>105% RFD: 105% > 110%>105% PV: 120% > 110%>105% ES: small-to-moderate	Eccentric loads (120%) produced the greatest improvements in pGRF, PP, and PV, while RFD peakedat load (105%), suggesting a load dependent trade off between explosive power and rapid force production.
Lloyd et al. (2022)	drop jump	Dumbbells	CON:BW ECC:15%+BW	-	jump height Contact time; RSI Peak CoM; displacement; mean con power; mean ecc power Immediate (Same rep)	In the AEL group, ground reaction force increased by 15% (p < 0.05), contact time decreased by 10% (p < 0.05), and jump height increased by 8% (p < 0.05). Hedges’g = 0.4–0.6	AEL significantly enhanced jump mechanics, reflected by increased force output, reduced ground contact time, and improved jump height.

Abbreviations: CMJ, countermovement jump; SJ, squat jump; AEL, accentuated eccentric loading; BM, body mass; TRT, traditional resistance training; CON, concentric; ECC, eccentric; RM, repetition maximum; RSI, reactive strength index; RFD, rate of force development; PP, peak power; PV, peak velocity; w, week; DJ, drop jump; MP, mean power; MV, mean velocity; MP, mean power; Com, Center of Mass.

**TABLE 2:** Chronic AEL training studies.

Study	Movement	Loading method	Protocol		Assessment	During	Results	Conclusions
			E	C				
			Load magnitude	Load magnitude		Sessions		
Sheppard et al. (2008)	CMJ	Dumbbells	Men ECC: 40 kg CON: BM Women ECC: 20 kg CON: B 2 × 5 AEL on all reps	Men ECC: BM CON: BM Women ECC: BM CON: BM 2 × 5	CMJ PV PF PP	3/5w 15	AEL Group: Significant improvements in displacement (+11%), peak velocity (+16%), and peak power (+20%) TRT Group: No significant changes in displacement (−2%), peak velocity (−3%), or peak power (−1%) d = 1.06–1.97 (large)	AEL elicited substantial enhancements in movement performance metrics, while TRT showed no meaningful gains. Results support the superior efficacy of AEL over traditional loading
Walker et al. (2016)	Leg press and leg extension	Weight releasers	Session 1 CON: 6RM ECC: 6RM+40% CON(120 Session 2 CON: 10RM ECC: 10RM+40%CON	Session 1 CON: 6RM ECC: 6RM Session 2 CON: 10RM ECC: 10RM	maximum isometric force Strength squat 1RM	2/5w 2/5w 20	Maximum Isometric Force AEL: +18% (g = 0.95); TRT: +11% (g = 0.45) Peak Eccentric Torque AEL: +10% (g = 0.6); TRT: no significant change	AEL led to greater and statistically significant improvements in both isometric force and eccentric torque compared to TRT, which showed limited or no effect
Friedmann et al. (2004)	Leg press and leg extension	Computer-driven	CON: 30% 1RM ECC: 70% 1RM 3 × 25 ea leg	CON: 30% 1RM ECC: 30% 1RM 3 × 25 ea leg	Strength squat 1RM	2/4w 8	TRT: non-statistically significant change AEL: +5%	AEL produced a modest performance gain, while TRT failed to elicit significant change
Friedmann-Bette et al. (2010)	leg extension	Computer-driven	CON: 8RM ECC: 1.9x8RM 5	CON: 8RM ECC: 8RM 6	1RM leg extension Squat jump	3/6w 18	Non-significant difference between groups AEL significantly greater than TRT	Despite non-significant differences between groups, AEL showed superior within-group gains compared to TRT.
Godard et al. (1998)	leg extension	Computer-driven	CON: 80% 1RM ECC: 120% 1RM	CON: 80% 1RM ECC: 80% 1RM	Strength (CON 1RM torque)	2/10w 20	Both Groups Performance increased in both AEL and TRT No significant difference observed Between Group Comparison	Both interventions led to improvements, but neither was statistically superior to the other
Hortobagyi et al. (2001)	Leg extension	Manual adjustment by coach	CON: 60% 1RM ECC: 100%–110% 1RM 5 × 10	CON: 60% 1RM ECC: 60% 1RM 5 × 12	Maximal isometric strength, maximal isokinetic strength, 3RM leg extension (CON and ECC)	7days 7	Eccentric 3RM AEL: +27%; TRT: +11% Concentric 3RM AEL: +27%; TRT: +26% Max Isometric Strength AEL showed additional gains (exact % not specified)	AEL elicited greater improvements in eccentric strength compared to TRT, while concentric strength gains were comparable between groups. AEL also demonstrated added benefit in isometric force
Yarrow et al. (2008)	Squat	counterbala-nce weight system	CON: 40% 1RM ECC: 100% 1RM 3 × 6	CON: 52.5% 1RM ECC: 52.5% 1RM 4 × 6	Strength Squat 1RM	3/5w 15	Squat Strength Both groups improved similarly (22%) Between-Group Compariso No significant difference observed	Comparable strength gains in squat across AEL and TRT; neither intervention proved superior.
Bridgeman et al. (2017a)	CMJ Squat jump	Dumbbells	CON: BM ECC: 20%BM session1: 5 × 6 session2: 5 × 10	CON: BM ECC: BM session1: 5 × 6 session2: 5 × 10	CMJ Squat jump Concentric PF Eccentric PF	2/3w 6	AEL Group improve observed in jump height, concentric strength, and eccentric peak force	AEL enhanced both explosive and maximal force capacities across concentric and eccentric phases
Bridgeman et al. (2020)	CMJ Squat jump	Dumbbells	CON: BW ECC: 20%BW	CON: BW ECC: BW	CMJ SJ Concentric PF Eccentric PF sprint COD (505)	2/4w 8	AEL Group significant increases in concentric PF (+13.2%), eccentric PF (+13.8%), CMJ (+13%), and DJ (+14.8%) Decreased sprint and COD time	AEL significantly improved strength and jump performance, while also enhancing speed and COD through reduced sprint and COD times
Douglas et al. (2018)	Squat	Weight releasers	session1 CON: 68%–72% 1RM ECC: 92%–98% 1RM 3 × 6 session2: CON: 77%–81% 1RM ECC: 106%–110% 1RM 4 × 4	session1 CON: 68%–72% 1RM ECC: 68%–72% 1RM 3 × 6 session2: CON: 77%–81% 1RM ECC: 77%–81% 1RM 4 × 4	Strength Squat 1RM 40 m sprint PV RSI	2s/8w 16	Slow AEL Improvements in squat 1RM (ES = 0.48), 40 m sprint time (ES = 0.28), and maximum velocity (ES = 0.52) Fast AEL Increased RSI	Slow AEL enhanced maximal strength and sprint performance, while fast AEL improved RSI velocity-specific adaptations
Munger et al. (2022), Munger, (2020)	Squat	Weight releasers	CON: 60%1RM ECC: 110% 1RM 4 × 5	CON: 85% 1RM ECC: 85% 1RM 4 × 5	Strength Squat 1RM CMJ	2/5w 10	AEL Increased 1RM and CMJ height (trivial to small)	AEL effectively enhanced both maximal strength and jump performance

Abbreviations: CMJ, countermovement jump; SJ, squat jump; AEL, accentuated eccentric loading; BM, body mass; TRT, traditional resistance training; CON, concentric; ECC, eccentric; RM, repetition maximum; pGRF, peak ground reaction force; RFD, rate of force development; PP, peak power; PV, peak velocity; RSI, reactive strength index; w, week; COD, change of direction; DJ, drop jump; MP, mean power; MV, mean velocity; MP, mean power; RSI, reactive strength index.

### 2.6 Risk of bias assessment

The methodological quality and risk of bias of the included studies were independently assessed using the Downs and Black checklist (Dow et al., 1998). This instrument evaluates four core domains: reporting quality, internal validity, external validity, and statistical power, across 25 items from the original scale. Two reviewers (Z.J.H. and Y.T.W.) independently scored each study, and any disagreements were resolved through discussion or adjudication by a third reviewer (X.Y.).

Consistent with prior systematic reviews, a quality percentage score was calculated for each study by dividing the total score by the maximum possible score and multiplying by 100. Based on this percentage, studies were categorized into three quality tiers: high quality (>66.7%, low risk of bias), moderate quality (50.0%–66.6%, moderate risk of bias), and low quality (<50.0%, high risk of bias). Studies classified as low quality were excluded from the final analysis to maintain overall methodological rigor.

Based on the Downs and Black assessment, all included studies–comprising nine acute studies and 11 chronic studies–adopted a controlled design. Notably, no study was categorized as high risk. A summary of methodological quality scores and risk-of-bias tiers is provided in Table 3, while the full itemized scoring breakdown for each study. Overall, the evidence base can be considered of moderate methodological quality.

**TABLE 3:** Methodological quality assessment using the Downs and Black checklist.

Study	Total score	Quality score (%)	Risk of bias tier
Aboodarda et al. (2013)	21	84%	High Quality (Low Risk)
Bridgeman et al. (2017b)	21	84%	High Quality (Low Risk)
Lloyd et al. (2022)	20	80%	High Quality (Low Risk)
Merrigan et al. (2020)	20	80%	High Quality (Low Risk)
Munger et al. (2017)	20	80%	High Quality (Low Risk)
Munger (2020)	20	80%	High Quality (Low Risk)
Sheppard et al. (2008)	20	80%	High Quality (Low Risk)
Wagle et al. (2018)	20	80%	High Quality (Low Risk)
Walker et al. (2016)	20	80%	High Quality (Low Risk)
Friedmann et al. (2004)	20	80%	High Quality (Low Risk)
Friedmann-Bette et al. (2010)	20	80%	High Quality (Low Risk)
Douglas et al. (2018)	17	68%	Moderate Quality (Moderate Risk)
Moore et al. (2007)	16	64%	Moderate Quality (Moderate Risk)
Yarrow et al. (2008)	16	64%	Moderate Quality (Moderate Risk)
Sheppard et al. (2007)	15	60%	Moderate Quality (Moderate Risk)
Aboodarda et al. (2014)	14	56%	Moderate Quality (Moderate Risk)
Bridgeman et al. (2017a)	14	56%	Moderate Quality (Moderate Risk)
Bridgeman et al. (2020)	14	56%	Moderate Quality (Moderate Risk)
Godard et al. (1998)	14	56%	Moderate Quality (Moderate Risk)
Hortobagyi et al. (2001)	14	56%	Moderate Quality (Moderate Risk)

Scores are based on a modified Downs and Black checklist (maximum 25 points). Quality tiers: High Quality (>66.7%), Moderate Quality (50%–66.6%), Low Quality (<50%).

### 2.7 Certainty of evidence (GRADE)

We evaluated the certainty of evidence using the GRADE approach for each outcome. Two reviewers independently rated risk of bias, inconsistency, indirectness, imprecision, and publication bias, with disagreements resolved by consensus. In the absence of a meta-analysis, GRADE judgments were applied to a structured narrative synthesis. In this context, inconsistency was attributed to heterogeneity in study designs, outcome measures, and conflicting effect directions; imprecision was linked to small sample sizes and wide or unreported confidence intervals; indirectness reflected differences in population, intervention, comparator, or outcomes; and publication bias was rated as undetected or unclear when the number of studies was insufficient. Summary of Findings tables are presented.

## 3 Results

### 3.1 Characteristics of included studies

**FIGURE 1:**
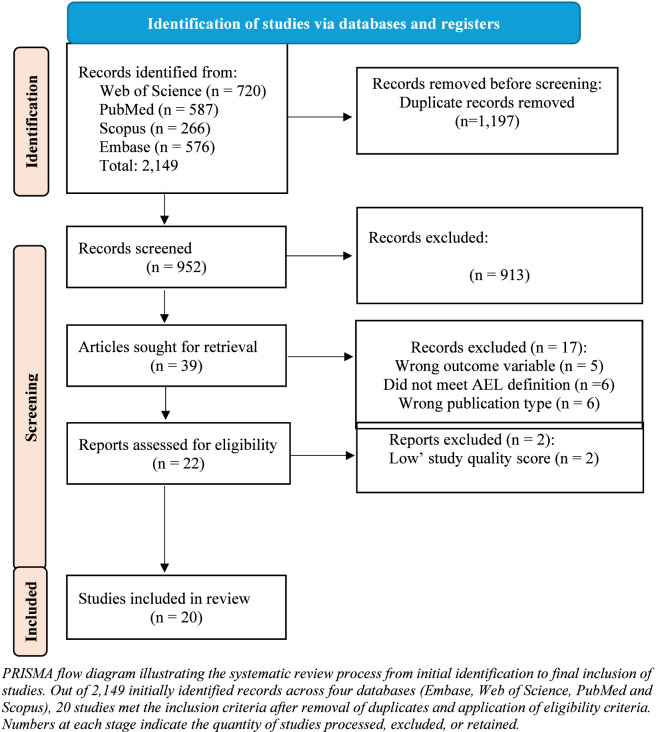
Flow diagram of the selection process.

An overview of the literature characteristics is presented in Table 4. A total of 2,149 articles were identified through electronic database searches, including Web of Science (n = 720), PubMed (n = 587), Scopus (n = 266), and Embase (n = 576). After removing 1,197 duplicate records, 952 unique records remained for initial screening based on titles and abstracts. Of these, 913 records were excluded due to irrelevance or failure to meet inclusion criteria. Subsequently, 39 full text articles were retrieved for detailed eligibility assessment. Seventeen articles were excluded at this stage for the following reasons: ineligible outcome variables (n = 5), ineligible intervention (n = 6), and non-eligible publication type (n = 6). The remaining 22 articles underwent methodological quality assessment, and two were excluded due to low methodological quality scores. Ultimately, 20 studies met all inclusion criteria and were included in the final systematic review. The complete study selection process is illustrated in Figure 1, with exclusion reasons and article numbers specified at each stage.

**TABLE 4:** Characteristics of the studies.

References	Sample					Study design
	Training status	Strength level (subjective description; years of RT experience; relative strength levels BM/1RM)	Size (M-F)	Age [Mean (SD)]	Mass [Mean (SD)]	
Sheppard et al. (2008)	Elite volleyball players	NR	16 (10–6)	21.8 (4.9)	83.7 (4.2)	Chronic: randomized controlled trial
Walker et al. (2016)	≥2years strength trainer	NR	28 (28–0)	22 (3.0)	76 (10)	Chronic: randomized controlled trial
Friedmann et al. (2004)	Untrained	No RT within 1 year	18 (18–0)	24.6 (3.4)	78.3 (4.6)	Chronic: Randomized Controlled Trial
Friedmann-Bette et al. (2010)	>1 year strength trainer	NR	25 (25–0)	24.4 (4.0)	80.06 (8.3)	Chronic: randomized controlled trial
Godard et al. (1998)	Strength trainer	NR	28 (16–12)	21.6 (2.4)	70.1 (12.3)	Chronic: randomized controlled trial
Hortobagyi et al. (2001)	Untrained	NR	20 (0–20)	20.9 (1.2)	61.3 (7.3)	Chronic: randomized controlled trial
Yarrow et al. (2008)	Untrained	NR	22 (22–0)	22.1 (0.8)	79.94 (2.9)	Chronic: randomized controlled trial
Bridgeman et al. (2017a)	≥2years Strength trainer	NR	8 (8–0)	26.3 (5.1)	82.94 (12.4)	Chronic: cross-sectional repeated measures design
Bridgeman et al. (2020)	Academy rugby players	1.65xBM squat	8 (8–0)	18.7 (1.0)	92.7 (11.6)	Chronic: randomized controlled trial
Douglas et al. (2018)	Academy rugby players	>1.7xBM squat	14 (14–0)	19.4 (0.8)	97.0 (11.6)	Chronic: randomized controlled trial
Munger, (2020)	Strength trainer	>1.7xBM squat	23 (23–0)	21.42 (3.1)	79.32 (10.9)	Chronic: randomized controlled trial
Aboodarda et al. (2014)	Rugby and Crossfit trainer	>2xBM squat	15 (15–0)	24.7 (5.7)	87.8 (10.5)	Acute: randomized repeated-measure design
Aboodarda et al. (2013)	>6 months strength trainer	NR	15 (15–0)	22.6 (5.3)	74.9 (4.1)	Acute: randomized repeated-measures design
Bridgeman et al. (2017b)	≥2years Strength trainer	>1.5xBM squat	12 (12–0)	25.4 (3.5)	84.1 (10.1)	Acute: repeated-measures design
Moore et al. (2007)	>6 months strength trainer	>1.5xBM squat	13 (13–0)	22.8 (2.9)	87.1 (11.8)	Acute: repeated-measures design
Sheppard et al. (2007)	Elite volleyball players	NR	11 (11–0)	18.9 (2.6)	91.4 (8.2)	Acute: randomized controlled trial
Wagle et al. (2018)	>1 year strength trainer	>1.5xBM squat	11 (11–0)	26.1 (4.1)	92.5 (10.5)	Acute: randomized repeated-measures design
Merrigan et al. (2020)	Strength trainer	>2xBM squat	21 (21–0)	24.0 (4.2)	85.5 (11.2)	Acute: repeated-measures crossover design
Munger et al. (2017)	>6 months strength trainer	>1.3xBM squat	20 (20–0)	23.8 (1.8)	83.49 (10.20)	Acute: randomized repeated-measures design
Lloyd et al. (2022)	rugby players >1 year strength trainer	NR	15 (15–0)	16.2 (1.0)	73.38 (9.9)	Acute: cross-over design

Abbreviations: NR, reported; BM, body mass; M, male; F, female; RT, resistance training; SD, standard deviation; 1RM, One-repetition maximum.

### 3.2 Risk of bias assessment

Based on the Downs and Black assessment (Dow et al., 1998), all included studies–comprising 9 acute studies and 11 chronic studies–adopted a controlled design, either randomized crossover trials or randomized controlled trials, and incorporated assessments of power or explosive performance during or after the intervention. Among the 20 studies evaluated, 8 (40%) were rated as having a low risk of bias, while the remaining 12 (60%) demonstrated a moderate risk of bias. Notably, no study was categorized as high risk. A detailed summary of each study’s methodological quality scores is provided in Table 3. Overall, the evidence base can be considered of moderate methodological quality.

### 3.3 Summary of findings

#### 3.3.1 Participants

A total of 20 studies published between 1998 and 2023 were included in this review, comprising 9 acute studies and 11 chronic training studies (Tables 1 and 2). These studies collectively tested AEL in over 400 participants, with sample sizes ranging from n = 8 (Bridgeman et al., 2020; Munger, 2020) to n = 28 (Godard et al., 1998) per group. The age of participants ranged from 18.7 ± 1.0 years (Bridgeman et al., 2020) to 26.1 ± 4.1 years (Wagle et al., 2018), while body mass ranged from 61.3 ± 7.3 kg (Hortobagyi et al., 2001) to 92.5 ± 10.5 kg (Wagle et al., 2018). The majority of participants were young male adults with some resistance training experience, although a few studies included female participants (Sheppard et al., 2008; Hortobagyi et al., 2001) or younger athletes (Lloyd et al., 2022). Three studies specifically recruited untrained participants (Friedmann et al., 2004; Hortobagyi et al., 2001; Yarrow et al., 2008), whereas the remainder involved moderately to highly trained individuals, many of whom were athletes or experienced trainees. Baseline strength levels, when reported, mostly fell within the range of a 1RM back squat equivalent to 1.3–2.0 × body mass, confirming that most participants were at least intermediate level trained. Such baseline characteristics suggest that the evidence primarily reflects outcomes in relatively trained populations, with limited generalisability to untrained or clinical cohorts. Across the included trials, standardized ES generally aligned with these percentage changes (Tables 1, 2). For maximal strength outcomes, within- and between-group ES were most often in the moderate-to-large range, whereas jump performance outcomes tended to show small-to-moderate effects. In contrast, ES for sprint and COD measures were typically small or trivial and often statistically unclear. Where reported, between-group effects usually favoured AEL over traditional loading for maximal strength, while the advantages for explosive and speed-related outcomes were less consistent.

### 3.4 Acute effects of AEL

The acute literature examines the immediate effects of AEL on exercise performance relative to TRT, with results summarized in Table 1. Performance variables are captured either within the same repetition by contrasting concentric power preceded by an eccentric overload with those recorded under normal loading, or during the repetitions and sets that follow the overload stimulus. These designs elucidate short-term neuromuscular responses, particularly post activation potentiation (PAPE), and provide empirical guidance for selecting eccentric intensities that maximize performance.

The immediate concentric response to AEL is strongly influenced by the magnitude of the eccentric overload. Moore et al. (2007) examined loaded jump squats at a 30% 1RM concentric load with different eccentric loads (30%, 50%, 80%, and 110% 1RM). They observed that peak concentric velocity was highest under the 110% eccentric condition, slightly above the velocity with no overload, whereas the 50% and 80% 1RM eccentric conditions produced slightly lower velocities (Moore et al., 2007). These results cannot indicate that an eccentric-to-concentric ratio of at least 1.1 is required to elicit acute performance enhancement, while moderate overloads may be insufficient or even detrimental, likely because they disrupt the timing of the SSC. Likewise, in dynamic bench press throws, researchers have reported that adding 105%–120% 1RM eccentrically *via* weight releasers increased subsequent concentric velocity and power output compared with no eccentric overload (Castro et al., 2020; Kristiansen et al., 2022).

Despite these positive findings in controlled lifts, excessive eccentric loading can impair acute performance in ballistic tasks. Taber et al. (2023) investigated loaded countermovement jumps performed with and without an additional 10–30 kg applied through weight releasers. Although the overload elicited higher eccentric braking forces, the propulsive impulse was reduced, which limited take off velocity. The authors suggested that forced deceleration of a large external mass disrupts the SSC and delays force transmission, thereby negating any potential performance benefit. These observations indicate that acute potentiation from AEL is more reliable in traditional resistance exercises or in repetitions following the overload than in the first concentric action of a maximal ballistic movement.

A different experimental approach examines whether a single supramaximal eccentric repetition can enhance subsequent submaximal repetitions within the same set. Merrigan et al. (2020), Merrigan et al. (2021) conducted a crossover study in which the first repetition of a back squat set incorporated a 120% 1RM eccentric load applied with weight releasers, while the following four repetitions were performed concentrically at 65% or 80% 1RM without additional overload. When the concentric load was 65% 1RM, repetitions two to five showed higher peak velocity and power than identical repetitions completed without the eccentric preload, confirming a PAPE effect (Merrigan et al., 2020). In contrast, at 80% 1RM the potentiation was negligible and manifested only as a small increase in peak force (Merrigan et al., 2020). These data indicate that the acute benefit of a heavy eccentric preload is expressed primarily when the ensuing concentric action is sufficiently fast, whereas heavier concentric loads, which elicit slower movement velocities, can obscure or counteract the potentiation through fatigue. The observation that velocity enhancement emerged from the second repetition onward further suggests that a brief recovery period is required for the potentiation to be realized. Taken together, these findings provide a rationale for extending the use of supramaximal eccentrics beyond a single set to explore their role as a conditioning activity before explosive tasks.

Building on this rationale, subsequent studies have tested supramaximal eccentric repetitions as a conditioning activity performed immediately before explosive tasks such as jumps or sprints. Two studies evaluated whether back squats incorporating supramaximal eccentric loads could serve as a conditioning activity before athletic testing (Tseng et al., 2021). Results were mixed: in one study, jump height improved when measured two to 3 min after AEL squats compared to a traditional squat warm up; in the other, no meaningful enhancement in sprint time was observed. These divergent outcomes likely reflect individual variability in responsiveness to PAPE and the influence of timing (Bridgeman et al., 2017b). Nonetheless, the broader pattern suggests that AEL can acutely stimulate the neuromuscular system in a manner that may enhance explosive performance, provided the loading protocol is well matched to the athlete’s physical profile. Specifically, stronger individuals with greater fatigue resistance may derive more benefit from this approach because they recover more quickly from the eccentric stimulus.

Across studies, AEL reliably elevates eccentric phase variables such as peak force and total mechanical work, and can enhance concentric phase performance when appropriately dosed. However, insufficient eccentric loading tends to produce negligible effects, whereas excessive overload can acutely impair output, particularly in ballistic movements. When used as a potentiating stimulus, the effectiveness of AEL depends on aligning the subsequent concentric load with the athlete’s neuromuscular profile and allowing adequate recovery time between the eccentric stimulus and the explosive action. These principles have direct implications for program design. For example, coaches may implement one or two heavy eccentric repetitions to potentiate lighter, high velocity efforts such as jumps or submaximal lifts, provided the transition period is sufficient and the subsequent movement is not overly fatiguing. Taken together, these acute trials indicate that AEL does not guarantee an immediate performance benefit; rather, the observed acute response depends on the balance between potentiation and fatigue. Protocols that align eccentric load and recovery interval with the athlete’s strength and power capacities are more likely to elicit a clear PAPE effect, whereas excessively heavy or poorly timed AEL exposures tend to yield neutral or even detrimental acute outcomes. Where reported, the acute enhancements in jump height, concentric velocity, or power under AEL were generally small-to-moderate in magnitude, with the largest ES observed when eccentric loads were set at 110%–120% of 1RM or 20%–30% of body mass.

### 3.5 Chronic effects of AEL

Across the included longitudinal studies lasting four to 10 weeks, reported gains in maximal strength such as one repetition maximum squat or leg press ranged from approximately 5% (Friedmann et al., 2004) to 27% (Hortobagyi et al., 2001), with most studies clustering between 9% and 22% (Walker et al., 2016; Wagle et al., 2017). Improvements in vertical jump height typically ranged from 4% to 13% (Sheppard et al., 2008; Bridgeman et al., 2020), while sprint time reductions were generally smaller, reaching up to 4% in some trials (Douglas et al., 2018; Bridgeman et al., 2020) but showing no significant change in others. Where standardized effect sizes or ES were reported, strength gains were typically associated with moderate to large effects, whereas improvements in jump and sprint performance were generally small to moderate. The most pronounced chronic adaptations were observed in well-trained who combined eccentrics at 110%–120% of concentric load with moderate to heavy concentric resistance.

#### 3.5.1 Maximal strength gains

Multiple investigations have compared changes in 1RM squat strength after training with AEL *versus* conventional loading. In resistance trained participants, AEL is at least as effective as traditional protocols and can, under certain prescriptions, produce superior adaptations.

Bridgeman et al. (2020) reported 15% gains with AEL *versus* 10% in controls, with moderate ES and greater improvements in reactive jump strength. Munger (2020) reported a 22% increase across both slow- and fast-tempo AEL, with tempo influencing adaptation type: slower eccentrics enhanced sprint speed, whereas faster eccentrics improved jump indices. Both AEL loading conditions stimulated greater strength gains than those observed with traditional loading strategies.

Walker et al. (2016) found similar 20%–22% increases after 10 weeks of AEL (110% eccentric, 60%–80% concentric) and traditional leg press and leg extension, and Hortobagyi et al. (2001) likewise reported no advantage in untrained participants (30% gains under both conditions). These findings suggest limited early phase benefit for novices but greater responsiveness in trained individuals.

Overall, AEL reliably enhances maximal strength to a degree comparable with conventional training and, under certain prescriptions, provides modestly larger gains, particularly in trained athletes using heavy concentric loads. Mechanistically, supramaximal eccentrics appear to augment force absorbing capacity and neural drive during lengthening, which may translate into higher concentric 1RM. Supporting this, Friedmann et al. (2004) observed isometric force increases of 18% with AEL *versus* 11% in controls, with eccentric torque gains unique to AEL. Collectively, these longitudinal data indicate that the standardized effects of AEL on maximal strength are generally moderate to large, reinforcing its efficacy as a stimulus for increasing lower-limb force capacity.

#### 3.5.2 Power and jump performance

Explosive lower body power, typically assessed through vertical jump metrics, is a primary outcome in chronic AEL studies. Bridgeman et al. (2020) found that AEL improved countermovement jump height by 8%, double the gain of the control group (4%), along with unique increases in eccentric rate of force development and peak force. Similarly, Sheppard et al. (2008) reported that 15 sessions of jump squats with a 40% body mass eccentric load, released before takeoff, produced greater improvements in loaded jump height than conventional body mass jumps.

Not all studies have demonstrated clear advantages of AEL for enhancing jump performance. In a 10 weeks leg-press intervention, Maroto-Izquierdo et al. (2023) compared a submaximal eccentric load of 90% 1RM with a supramaximal eccentric load of 120%, while maintaining the concentric phase at 30% 1RM. Both loading strategies increased lower-limb strength and countermovement-jump performance, yet the difference between the two conditions in jump height was small and statistically uncertain. Similar outcomes were reported by Friedmann-Bette et al. (2010), who applied AEL during leg-extension exercises and noted greater improvements within the AEL group but no significant advantage over traditional resistance training when groups were compared directly. Collectively, these findings indicate that progressively increasing the eccentric overload or relying on machine-based exercise models does not consistently result in superior adaptations in jump performance compared with lower eccentric magnitudes or conventional loading schemes. Across these trials, standardized effects on jump height and power clustered predominantly in the small-to-moderate range, with larger effects emerging when supramaximal eccentric loads or 20%–30% body-mass overloads were employed.

#### 3.5.3 Speed and change-of-direction performance

Empirical research examining the chronic effects of AEL on sprint and COD performance remains limited, yet two investigations provide the most direct evidence to date. Bridgeman et al. (2020) applied a jump-specific eccentric-overload protocol using dumbbells equivalent to 20% BW during the eccentric phase while maintaining body mass loading concentrically. Over 4 weeks of training, the AEL group demonstrated clear reductions in sprint time and COD (505 test) time compared with baseline, indicating that moderate eccentric loading applied in ballistic, task-specific movements can enhance both acceleration and multidirectional speed. Douglas et al. (2018) implemented an 8-week squat program with weight releasers and contrasted two eccentric-velocity conditions. The slower-velocity loading, performed with eccentric intensities between 92% and 98% 1RM, 40-m sprint time decreased, and maximal sprinting velocity increased, whereas the faster-velocity condition produced no measurable advantage for sprinting or COD (505 test).

These findings suggest that improvements in sprint and COD performance capacities following eccentric-overload training are most likely to occur when the eccentric stimulus is moderate in magnitude, movement-specific, and executed under controlled-velocity conditions. Excessively rapid or heavy eccentric actions appear less effective for transferring strength gains into enhanced movement speed.

### 3.6 AEL training parameters

#### 3.6.1 AEL implementation

Across the twenty trials, six methods were used to impose greater load during the eccentric than concentric phase. The most common was weight releasers, reported in seven studies (Douglas et al., 2018; Walker et al., 2016; Munger, 2020; Moore et al., 2007; Wagle et al., 2018; Merrigan et al., 2020; Munger et al., 2017), which attached to a barbell during descent and disengaged at the lowest point. Six studies used hand held dumbbells released at the eccentric-concentric transition in jumps or squats (Sheppard et al., 2008; Bridgeman et al., 2017a; Bridgeman et al., 2020; Bridgeman et al., 2017b; Lloyd et al., 2022). Three employed computer controlled devices such as isokinetic dynamometers or motorised leg press machines that increased resistance during the eccentric phase (Bridgeman et al., 2017b; Moore et al., 2007; Sheppard et al., 2007). Two used elastic bands to add eccentric tension or reduce concentric load (Aboodarda et al., 2014; Aboodarda et al., 2013). A counter weighted pulley system (Yarrow et al., 2008) and manual plate manipulation (Godard et al., 1998) were each described once. All protocols imposed higher eccentric than concentric loads, thereby meeting the operational definition of AEL.

#### 3.6.2 Loading magnitudes

Eccentric intensity relative to 1RM varied substantially across studies. In supramaximal protocols, the eccentric load exceeded the concentric 1RM. The most common prescriptions were 105%, 110%, and 120% of 1RM. Four trials implemented 120% (Walker et al., 2016; Godard et al., 1998; Merrigan et al., 2020; Munger et al., 2017), three used 110% [15, 27, 29, and two applied 105% (Douglas et al., 2018; Munger, 2020). Paired concentric loads ranged from 40% to 90% 1RM: lighter ranges of 30%–60% emphasised velocity (Friedmann et al., 2004; Hortobagyi et al., 2001; Yarrow et al., 2008; Munger, 2020), whereas heavier ranges of 80%–90% targeted maximal strength (Walker et al., 2016; Godard et al., 1998; Merrigan et al., 2020; Munger et al., 2017). Nine trials adopted submaximal eccentric overload, where the eccentric load remained below concentric 1RM but exceeded the concentric phase. Typical strategies included adding 20%–30% of body mass to the eccentric phase of jump tasks (Aboodarda et al., 2014; Aboodarda et al., 2013) or prescribing 70%–90% eccentric loads with 30%–60% concentric loads (Friedmann et al., 2004). In plyometric protocols, participants performed unloaded concentric jumps while holding additional dumbbells of 10%–30% body mass during descent (Sheppard et al., 2008; Bridgeman et al., 2017a; Bridgeman et al., 2017b; Lloyd et al., 2022). One study suggested that 20% body mass overload yielded better subsequent jump performance than 10%–30%, pointing to a possible optimal range around 20% (Bridgeman et al., 2017a).

Overall, supramaximal eccentric intensities of 105%–120% 1RM and submaximal overloads of 20% body mass are most frequently studied. Heavier eccentric prescriptions appear to favour maximal strength, whereas lighter submaximal loads are particularly effective for enhancing explosive plyometric performance.

#### 3.6.3 Intervention duration

The duration of longitudinal AEL interventions varied considerably, ranging from 1 to 10-week and yielding a total of 6–20 training sessions across studies. Most programmes were conducted at a frequency of 2–3 sessions per week. Representative examples include six sessions across 3 weeks (Bridgeman et al., 2017a), 15 sessions delivered over 7–8 weeks (Sheppard et al., 2008), 16 sessions during an 8–week period (Douglas et al., 2018), and 20 sessions across 10 weeks (Walker et al., 2016). In contrast to these multi week protocols, acute investigations focused on single session effects within a workout, directly comparing immediate performance outcomes under AEL with those achieved through symmetrical loading.

#### 3.6.4 Outcome measures

Performance outcomes were grouped into four main domains. These included (i) jump performance, assessed in 10 studies primarily through countermovement or drop jump height, with occasional inclusion of squat jump height (Bridgeman et al., 2017a; Aboodarda et al., 2014). (ii) Maximal strength, typically measured as 1RM squat or leg press strength in eight studies (Walker et al., 2016; Friedmann et al., 2004; Friedmann-Bette et al., 2010; Godard et al., 1998; Hortobagyi et al., 2001). (iii) Kinetic and kinematic variables such as peak and mean force, velocity and power, recorded in 11 studies, particularly in acute trials that analyzed each repetition within the AEL set. (iv) Sprint or change of direction ability, evaluated in a smaller subset using 30 m sprint times or shuttle tests (Douglas et al., 2018; Bridgeman et al., 2020). However, it is important to note that the shuttle test includes substantial linear running components and involves maneuvering around cones rather than sharp deceleration and re-acceleration. Therefore, its outcomes may reflect general running speed or maneuverability rather than isolated COD ability. In addition to these performance outcomes, a few studies incorporated mechanistic assessments. Two investigations collected muscle biopsy samples to examine fiber type mRNA expression (Aboodarda et al., 2014; Aboodarda et al., 2013), while one study monitored endocrine responses such as growth hormone and insulin like growth factor^−1^ to compare AEL with conventional loading. Baseline comparability was ensured across groups in controlled trials, and crossover studies included familiarization sessions to minimize learning effects. The findings from these varied protocols are synthesized in the subsequent sections to clarify how implementation choices modulate the acute and chronic effects of AEL.

## 4 Discussion

The accumulated evidence indicates that AEL is an efficacious method for improving strength and power, thereby corroborating its mechanistic rationale. Acute applications of AEL can elicit transient enhancements consistent with PAPE when eccentric intensity and recovery interval are appropriately configured (Seitz et al., 2014). Over longer training periods, programmes that integrate AEL consistently produce gains in maximal strength and explosive performance that are at least comparable to, and in some cases greater than, those observed with traditional symmetrical loading. Consequently, this systematic analysis extends previous narrative accounts (Nuzzo et al., 2023a) by providing empirically supported evidence that the benefits of AEL are observable across diverse participant cohorts, exercise modalities, and study designs. Viewed through this applied lens, the present synthesis connects mechanistic insights to programming decisions on load, timing, and frequency, and clarifies both the performance contexts in which AEL is advantageous and the current boundaries of evidence.

### 4.1 AEL *versus* TRT: programming contrasts and mechanisms

Building on prior accounts, we frame the review around the programming decision between AEL and TRT and integrate methodological contrasts with mechanism anchored interpretation. Methodologically, we define AEL as an eccentric intensity that exceeds the paired concentric load within the same repetition, and we evaluate comparative studies according to four methodological safeguards: matching total volume load, ensuring adequate exposure through multi week training blocks rather than isolated sessions, using comparable outcome protocols such as the same squat or jump tests, and considering training status as a moderator (Cormie et al., 2011; Walker et al., 2016; Douglas et al., 2017b; Wagle et al., 2017). Mechanistically, we interpret effects through properties that are not fully stimulated by symmetrical loading. These include a greater force capacity during lengthening which is on average about 40% higher than during shortening, preferential recruitment of high threshold motor units under high tension, residual force enhancement, and potential adaptations of series elastic elements such as tendon stiffness and fascicle–tendon interactions (Nuzzo et al., 2023b; Duchateau and Enoka, 2016). Viewed through this integrated lens, consistent practice guidelines emerge across studies. The most effective prescriptions typically involve eccentric intensities of 110%–120% 1RM in multi-joint lifts, or an additional 10%–30% of body mass in eccentric jump drills. At the same time, the evidence remains less certain for outcomes such as sprinting and change of direction, even though moderate certainty advantages for maximal strength are supported (Walker et al., 2016; Douglas et al., 2017b; Seitz and Haff, 2016; Wagle et al., 2017; Munger, 2020). In summary, our contribution is a programming centred synthesis that delineates the AEL–TRT contrast with explicit methodological criteria, links outcomes to mechanistic rationale, and provides actionable dosing guidance while defining the boundaries of inference.

### 4.2 Acute responses *versus* chronic adaptations: time course and certainty

A clear distinction emerges between the time courses of AEL adaptations. Acute performance enhancements occur only under specific conditions, particularly in well-trained athletes and when the eccentric load is sufficiently high (Merrigan et al., 2021), whereas chronic improvements are reported more consistently, even when immediate potentiation is absent (Gu et al., 2025). This pattern suggests that the lack of an acute gain during a single AEL exposure does not preclude meaningful long-term adaptation (Cormie et al., 2011). High eccentric tension may initiate muscular and neural remodeling across successive sessions (Walker et al., 2016), even if transient fatigue masks short-term performance (Bridgeman et al., 2017a). Conversely, when acute potentiation is observed, the stimulus is likely well-dosed and, if repeated over several weeks, can lead to greater strength and power gains (Douglas et al., 2018). Collectively, the acute and chronic findings highlight the dual role of AEL: it serves both as an immediate neuromuscular activator and as a progressive overload stimulus for long-term athletic development (Chae et al., 2024). Relative strength may further moderate responsiveness to AEL. For example, a back squat strength of 1.3–2.0 x body mass spans a broad range of training status, and athletes with higher relative strength may be better positioned to benefit from eccentric overload. However, few included studies reported outcomes stratified by relative strength, limiting firm conclusions.

### 4.3 Low load *versus* supramaximal: dosing and fatigue cost

At the lower end of the eccentric loading continuum, adding 10%–30% of body mass can acutely improve SSC efficiency and concentric output. The extra load increases braking force and muscle tendon stretch, thereby storing more elastic energy and elevating neuromuscular activation before take off. Empirical findings support this mechanism. In high performance athletes, countermovement jumps performed with a 20 kg eccentric overload released immediately before propulsion produced a 4% increase in jump height and a 9% increase in peak power compared with body mass jumps (Sheppard et al., 2008). In adolescents, adding 15% of body mass during drop jumps similarly enhanced jump height and produced moderate gains in eccentric braking and concentric impulse. These observations suggest that modest eccentric overloads, when precisely timed, can meaningfully augment explosive performance without the fatigue typically associated with supramaximal protocols.

Chronic exposure to low load AEL can also yield substantial gains in explosive performance. Over several weeks, the cumulative stimulus of repeated yet manageable eccentric overload elicited adaptations that exceeded those from traditional plyometric training. Sheppard et al. (2007) showed that 5 weeks of countermovement jump training with a 20 kg eccentric overload increased vertical jump displacement by 11%, whereas a control group performing body mass jumps showed a slight decline; peak power rose 20% under AEL but only 1% in controls. These findings confirm the efficacy of modest eccentric overload for improving power and jump height in trained athletes. Practical application, however, should account for experience and fatigue tolerance. Novice or youth athletes require sound landing mechanics before introducing AEL, as even light overload alters force distribution. Coaches should monitor markers such as muscle soreness and creatine kinase activity, which can peak 24 h after a 20% body mass overload (Merrigan et al., 2022; Hodgson et al., 2005). Although milder than supramaximal responses, progressive exposure and recovery remain essential, especially during early sessions. When appropriately supervised, low load AEL is well tolerated; for instance, an adolescent study reported no increase in landing forces with a 15% overload (Lloyd et al., 2022), Thus, with careful progression, light eccentric overload can safely improve jump height, power output, and SSC function.

At the high load end, supramaximal AEL prescribes eccentric intensities of 105%–120% 1RM. Leveraging the higher force capacity during lengthening (Merrigan et al., 2020), this approach provides a high tension stimulus that can increase neural drive, recruit high threshold motor units, and load the muscle tendon complex beyond symmetrical training. Comparative trials have demonstrated clearer advantages at supramaximal doses. In a 10week leg press study, 120% *versus* 90% eccentric loading (with a concentric load of 30% 1RM) produced greater improvements in 1RM (16%) and vertical jump height (10%–13%) (Maroto-Izquierdo et al., 2023). In well-trained athletes, incorporating 105%–110% 1RM eccentrics into multi-joint lifts has produced equal or greater strength gains than traditional loading and superior eccentric and isometric strength, helping to overcome plateaus (Walker et al., 2016).

Given the substantial mechanical stress, supramaximal AEL should be reserved for athletes with sufficient strength and technical proficiency and introduced gradually at low volumes. Safe application typically requires weight releasers or trained spotters. Early exposures may cause significant soreness and creatine kinase elevations, so longer recovery and monitoring for signs of overreach are recommended (Proske and Morgan, 2001). Weekly frequency is usually limited to one session. When applied carefully, heavy AEL provides a powerful stimulus for breaking plateaus and inducing neuromuscular adaptations beyond those achieved with conventional training.

### 4.4 Hypertrophy *versus* performance transfer: outcomes and boundaries

Expectations regarding hypertrophy and transfer should remain measured. While supramaximal loading introduces a novel strength stimulus, short-term muscle hypertrophy does not consistently exceed that observed with traditional resistance training. In well-trained participants completing 8–10 weeks of eccentric overload, increases in muscle cross sectional area have generally matched those from conventional protocols, typically 10%–15% (Walker et al., 2016). In Walker’s trial, both AEL and traditional groups achieved significant hypertrophy of the knee extensors with no difference between them. Similarly, Maroto-Izquierdo et al. (2023) reported comparable gains in lean mass under 120% and 90% eccentric conditions, despite the strength advantage of the heavier load. These findings suggest that supramaximal AEL is not required for hypertrophic adaptation; substantial growth can be achieved with moderate loads performed to high effort. The primary value of heavy eccentric work likely lies in its unique neural and mechanical demands, which may augment strength and power beyond changes in muscle size. Coaches may reserve supramaximal AEL for phases targeting maximal strength or for athletes who have plateaued with conventional methods. By integrating low load ballistic AEL and heavy overload AEL within periodised programmes, practitioners can take advantage of their complementary benefits: lighter loads enhance explosive power and stretch-shortening cycle efficiency with minimal fatigue, whereas heavier loads drive maximal strength but impose greater technical and recovery demands. When applied carefully, these strategies expand the range of effective stimuli available to improve athletic performance more safely and effectively than symmetrical resistance training alone.

Although the present review focused on lower-body resistance exercises, similar principles appear to apply to upper-limb conditioning activities. In a recent systematic review and meta-analysis on post-activation performance enhancement in the bench press throw, Krzysztofik et al. (2021) reported small-to-moderate improvements in upper-limb power following heavy bench press conditioning sets, with the magnitude of PAPE being moderated by conditioning load, rest interval and training status. These determinants closely mirror those identified in the current synthesis of lower-body AEL studies, where eccentric intensity, concentric loading and recovery duration influence whether an acute response is positive, neutral or negative. However, relatively few studies have implemented true upper-limb AEL configurations (eccentric loads exceeding concentric loads within each repetition), and further work is needed to establish whether the benefits observed in lower-body AEL can be generalised to pushing and throwing movements in the upper body.

Our conclusions reflect the certainty of evidence. Benefits for maximal strength are supported with moderate certainty, whereas evidence for explosive performance is low and for sprint or change of direction very low, largely due to small sample sizes, protocol heterogeneity, and imprecision. Future research should prioritise standardised protocols, larger randomised trials, and harmonised outcome definitions to enable robust pooled estimates and narrower certainty ranges.

## 5 Conclusion

When delivered with clearly defined eccentric parameters, AEL enhances lower body maximal strength and explosive performance. The most consistent benefits occur at eccentric intensities of 110%–120% of concentric 1RM or with an additional 10%–30% of body mass in jump drills. However, evidence for transfer to sprint and change of direction performance remains limited and context dependent. Given the heterogeneity of protocols and the small number of high quality trials, these conclusions should be interpreted with caution. Future work should prioritise standardisation of protocols, larger randomised trials, and clearer dose-response analyses to refine practical guidance.

## 6 Limitations and future directions

Current evidence on AEL is highly heterogeneous in eccentric intensity, concentric comparators, exercise modes, outcome definitions, and total training volume. This variability limits comparability across studies and prevents firm dose-response guidance. Samples were narrow, dominated by healthy young men, while women, adolescents, and older adults remain under represented. Most interventions were short, typically fewer than 10 weeks, leaving durability, long-term safety, and potential training plateaus unclear. Longer trials extending beyond 6 months are therefore required. The review was restricted to English language publications to ensure accurate extraction and coding of detailed training prescription variables and outcomes. This decision may have introduced language bias and led to the omission of relevant non-English studies. Readers should interpret the present synthesis with this limitation in mind.

Future research should standardise eccentric-to-concentric load ratios to establish outcome specific targets for strength, power, and tendon adaptations. Mechanistic studies using imaging and electrophysiological methods should track changes in muscle architecture, fibre type expression, and neural drive over time. Sport-specific trials that integrate AEL into relevant skills, including upper body tasks and change of direction drills, are needed to test external validity. Finally, stratified analyses by sex, age, and training status will enable more tailored and evidence-based prescriptions across athletic and clinical populations.

## Data Availability

The original contributions presented in the study are included in the article/supplementary material, further inquiries can be directed to the corresponding author.
